# Distribution of pathogenic variants in the *CFTR* gene in a representative cohort of people with cystic fibrosis in the Kingdom of Bahrain

**DOI:** 10.1007/s00438-024-02119-4

**Published:** 2024-05-14

**Authors:** Osama A. Karim Majed, Fatema Osama Majed, Nabeel Jasim Almoamen, Husain Baqer Alsatrawi, Salma Dawood Shehabi, Jana Hrbková, Malgorzata Libik, Milan Macek

**Affiliations:** 1 Salmaniya Medical Complex, Rd. No. 2904, Manama, Kingdom of Bahrain; 2 Royal University of Surgeons in Ireland, Medical University of Bahrain, Al Sayh Muharraq Governorate, Busaiteen, Kingdom of Bahrain; 3 Department of Pediatrics, University Hospital in Coventry and Warwickshire, Coventry, United Kingdom; 4 Department of Biology and Medical Genetics, 2nd Faculty of Medicine and Motol University Hospital, Charles University, Prague, Czechia

**Keywords:** Bahrain, Cystic fibrosis, *CFTR* gene, CFTR modulator therapy (CFTRm), Mutations, Sequencing

## Abstract

**Background:**

Cystic fibrosis (CF) is a rare multi-systemic recessive disorder. The spectrum and the frequencies of *CFTR* mutations causing CF vary amongst different populations in Europe and the Middle East. In this study, we characterised the distribution of CF-causing mutations (i.e. pathogenic variants in the  *CFTR* gene) in a representative CF cohort from the Kingdom of Bahrain based on a three-decade-long analysis at a single tertiary centre. We aim to improve CF genetic diagnostics, introduce of CF neonatal screening and provide CFTR modulator therapy (CFTRm).

**Methods:**

*CFTR* genotyping  and associated clinical information were drawn from a longitudinal cohort. We sequenced 56 people with CF (pwCF) that had one or both *CFTR* mutations unidentified and carried out comprehensive bioinformatic- and family-based segregation analyses of detected variants, including genotype–phenotype correlations and disease incidence estimates. The study methodology could serve as a basis for other non-European CF populations with a high degree of consanguinity.

**Results:**

Altogether 18 CF-causing mutations  were identified, 15 of which were not previously detected in Bahrain, accounting for close to 100% of all population-specific alleles. The most common alleles comprise c.1911delG [2043delG; 22.8%], c.2988+1G > A [3120+1G>A; 16.3%], c.2989-1G>A [3121-1G>A; 14.1%], c.3909C>G [N1303K; 13.0%], and c.1521_1523delCTT [p.PheF508del; 7.6%]. Although the proportion of 1st cousin marriages has decreased to 50%, the frequency of homozygosity in our pwCF is 67.4%, thereby indicating that CF still occurs in large, often related, families. pwCF in Bahrain present with faltering growth, pancreatic insufficiency and classical sino-pulmonary manifestations. Interestingly, two pwCF also suffer from sickle cell disease. The estimated incidence of CF in Bahrain based on data from the last three decades is 1 in 9,880 live births.

**Conclusion:**

The most commonCF-causing  mutations in Bahraini pwCF were identified, enabling more precise diagnosis, introduction of two-tier neonatal screening and fostering administration of CFTRm.

## Introduction

Cystic Fibrosis (CF; MIM: 219700) is a multi-system autosomal recessive rare disease caused by pathogenic, ‘CF-causing’, variants (henceforward mutations) in the Cystic Fibrosis Transmembrane Conductance Regulator (*CFTR*; MIM: 602421) gene (Riordan et al. 1998). More than 2,000 *CFTR* variants have been identified thus far (“CFTR1 database”—www.genet.sickkids.on.ca) with their type and frequency being variable in different populations (Boek 2020).

In the Middle East, the incidence of CF is estimated to range between 1 in 2000 and 1 in 5800 live births (Banjar and Angyalosi 2015). More than three decades ago, a retrospective study reported the incidence of CF in Bahrain as 1 in 7700 live births (Al Arrayed and Abdulla 1996), whilst a later study rendered an update of 1 in 5800 live births (Al-Mahroos 1998). The overall disease incidence reported in the latter study is likely biased since it was based on clinically diagnosed pwCF with a “severe” CF phenotype with most cases having early disease presentation due to faltering growth (previously termed as failure-to-thrive), pancreatic insufficiency and severe respiratory symptoms associated with colonisation of the lung by *P. aeruginosa*. Moreover, the lower number of identified pwCF together with the higher degree of consanguinity in Bahrain could further skew the final incidence estimates. Population-wise, most cases were of Bahraini origin, with several exceptions comprising pwCF of e.g. Saudi, Syrian and Persian origins (Al-Mahroos 1998; Eskandarani 2002).

Importantly, a particularly high rate of consanguinity (77%) was found amongst Bahraini CF families which could have augmented the frequency of several *CFTR* mutations. This specific population genetic aspect was reflected in commonly observed homozygosity in the most recent Bahraini report that however analysed a rather small cohort. Three common mutations were identified—p.Gln637HisfsX26 (legacy nomenclature 2043delG), p.His139Leu (H139L) and p.Phe508del in decreasing order of their frequencies (Eskandarani 2002).

This study aims to report basic demographic characteristics, clinical features and the distribution of CF-causing mutations in a representative group of Bahraini pwCF registered at the tertiary national CF centre at the Salmaniya Medical Complex (SMC), divided according to their population origin. Furthermore, the current study supersedes previously published limited reports, both in terms of the representativeness of the studied CF population and the overall number of pwCF examined by accounting for cases diagnosed over the last three decades. Furthermore, molecular genetic examination of the *CFTR* gene by massively parallel sequencing was complemented by copy number variation (CNV) analysis, including variants located at pre-selected *CFTR* intronic sites.

We hope that this study will provide a basis for improving diagnostics and clinical management, including the introduction of *CFTR* variant-specific therapies, such as CFTR modulator therapy (CFTRm) and facilitating the introduction of nationwide neonatal screening for CF in Bahrain.

## Patients and methods

This study consists of two complementary parts comprising the (a) retrospective compilation of demographic characteristics, clinical and laboratory features of CF and *CFTR* genotyping results drawn from SMC medical records followed by (b) prospective examination of living pwCF where one of both *CFTR* alleles (i.e. in *trans*) remained unidentified. pwCF selected for both retrospective analyses and prospective genetic examinations had to unambiguously comply with established diagnostic criteria of the classical form of the disease (Farrell et al. 2017). Consanguinity in tested CF families was analysed based on medical and/or pedigree records.

Initially, a total of 56 electronic medical records of clinically diagnosed pwCF registered at SMC between January 1990 and January 2020 had been comprehensively reviewed and 21  pwCF with previously identified CF-causing mutations on both parental chromosomes were noted. Genetic testing in pwCF who already had positive results was mostly carried out at a commercial laboratory (e.g. at Centogene.com, Germany; data available upon request). In addition, to provide a full picture of cases in the SMC database, we also included two pwCF with an atypical form of the disease who did not fulfil all diagnostic criteria for the classical form of CF. One pwCF had sweat chloride concentrations repeatedly below 60 mM, whilst the other was an infant who died from intestinal perforation before we could carry out complete genotyping (Table 1). However, due to unclear diagnosis, these detected variants were not considered in Table 2 to avoid bias since our primary focus is on classical forms of the disease. All clinical and laboratory data obtained through retrospective review were securely stored and reviewed at SMC by the authors.

**Table 1:** Demographic Data

	Bahraini origin	Non-Bahraini origin	Total (%)
Number of pwCF	42	14	56
Consanguinity %^a^	42.9%	72.7%	50.0%
Male:Female	28:14	4:10	Males: 32 (57.1%) Females: 24 (42.9%)
Mean age (years) ± SD (min–max)^b^	12.9 ± 6.9 (1.7—28.7)	7.6 ± 5.3 (1.7–23.1)	11.4 ± 6.9 (1.7—28.7)
Number of pwCF ≥ 18 years old (%)^b^	7 (20.6%)	1 (7.7%)	8 (17.0%)
Number of deaths (%)	8 (19.0%)	1 (7.1%)	9 (16.1%)
Mean age at death ± SD (min–max)	12.5 ± 4.4 (4.0–20.2)	16.5 (16.5)	12.9 ± 4.3 (4.0–20.2)
Mean age at diagnosis (months)^c^	15.1 ± 22.1 (1–108 months)	16.0 ± 22.8 (1–72 months)	15.9 ± 22.3 (1–108 months)
Median age at diagnosis (months)^c^	5.5 ± 16.4	6 ± 22.8	6 ± 18.3
Mean BMI (kg/m^2^) ± SD (min–max)^d^	15.3 ± 2.8 (12.2–24.9)	14.5 ± 1.8 (11.6–18.8)	15.1 ± 2.7 (11.6–24.9)
Mean sweat chloride test (mmol/L) ± SD (min–max)^e^	113.3 ± 12.9 (85–135)	102.2 ± 13.3 (73–116)	110.6 ± 13.9 (73–135)
Chronic *P. aeruginosa* lung colonisation (yes: no) (%)^f^	34:4 (89.5%)	9:2 (81.8%)	43 (87.8%)
Pancreatic insufficiency (%)	40 (95.2%)	14 (100.0%)	54 (96.4%)
CF-related diabetes mellitus (%)	4 (9.5%)	1 (7.1%)	5 (8.9%)
Concomitant non-CF-related diseases: Sickle-cell disease (%)	2 (4.8%)	–	2 (3.6%)
pwCF undergoing lung transplantation	1 (2.4%)	–	1 (1.8%)

^a^Consanguinity rate does not include 4 CF families as they were not reachable

^b^Age calculations excluded deceased pwCF

^c^Diagnosis age is for 53/56 pwCF

^d^The BMI results do not include 8 pwCF since they did not have recent BMI records

^e^The sweat chloride test results are for 46 pwCF. The remaining pwCF had sweat chloride tests over 60mM to confirm  their diagnosis; however, their results may have been lost due to a change in our medical case-filing system at SMC

^f^*P. aeruginosa* results are for 48 pwCF only. The percentages only reflect the pwCF who have undergone sputum culture or deep tracheal aspiration to test for *P. aeruginosa*

**Table 2:** Type and Frequency of *CFTR* Mutations in Bahrain

*CFTR* gene position	Mutation [Legacy name and/or Protein change]	Number of pwCF^a^	Number of pwCF that are consanguineous (Freq%)^b^	Number of pwCF as per country origin	Number of *CFTR* alleles for all pwCF (Freq%)^c^	Number of *CFTR* alleles after exclusion of siblings (Freq%)^d^	Number of pwCF homozygous for the *CFTR* allele (after exclusion of siblings)^e^	Number of pwCFwith compound heterozygosity for the *CFTR* allele (after exclusion of siblings)^e^
Exon 14	c.1911delG [2043delG;Gln637fsX662]	16	11 (68.8%)	Bahraini: 11	25 (22.3%)	21 (22.8%)	7	7
				Non-Bahraini: 5 Syrians				
Intron 18	c.2988+1G>A [3120+1G >A; IVS18+1G>A]	13	6 (46.2%)	Bahraini: 11	19 (16.9%)	15 (16.3%)	4	7
				Non-Bahraini: 2 Jordanians				
Intron 18	c.2989-1G>A [3121-1G>A; IVS18-1G>A]	9	4 (44.4%)	Bahraini: 9	15 (13.4%)	13 (14.1%)	5	3
				Non-Bahraini: 0				
Exon 24	c.3909C > G [N1303K; p.Asn1303Lys]	6	2 (33.3%)	Bahraini: 5	11 (9.8%)	12 (13.0%)	6	-
				Non-Bahraini: 1 Palestinian				
Exon 11	c.1521_1523delCTT [p.Phe508del]	7	5 (71.4%)	Bahraini: 6	13 (11.6%)	7 (7.6%)	3	1
				Non-Bahraini: 1 Pakistani				
Exon 2 and flanking intron regions	c.54-1161_164+1603del2875	3	3 (100%)	Bahraini: 1	6 (5.4%)	4 (4.3%)	2	-
				Non-Bahraini: 2 Jordanians				
Exon 11	c.1397C>G [S466X (TAG); p.Ser466X]	3	0 (0%)	Bahraini: 3	4 (3.6%)	4 (4.3%)	1	2
				Non-Bahraini: 0				
Exon 11	c.1418delG [1548delG; p.Gly473GlufsTer54]	2	1 (50%)	Bahraini: 1	3 (2.7%)	3 (3.3%)	1	1
				Non-Bahraini: 1 Yemeni				
Intron 6	c.743+2 T>C	2	2 (100%)	Bahraini: 2	4 (3.6%)	2 (2.2%)	1	-
				Non-Bahraini: 0				
Exon 12	c.1647 T>G [S549R(T>G); p.Ser549Arg]	2	1 (50%)	Bahraini: 2	2 (1.8%)	2 (2.2%)	-	2
				Non-Bahraini: 0				
Exon 14	c.2052dupA [p.Gln685ThrfsX4]	1	1 (100%)	Bahraini: 1	2 (1.8%)	2 (2.2%)	1	-
				Non-Bahraini: 0				
Intron 3	c.274-1G>A [406-1G>A]	1	0 (0%)	Bahraini: 0	1 (0.9%)	1 (1.1%)	-	1
				Non-Bahraini: 1 Indian				
Exon 3	c.223C>T [R75X; p.Arg75Ter]	1	0 (0%)	Bahraini: 0	1 (0.9%)	1 (1.1%)	-	1
				Non-Bahraini: 1 Indian				
Exon 4	c.416A>T [H139L; p.His139Leu]	1	0 (0%)	Bahraini: 1	1 (0.9%)	1 (1.1%)	-	1
				Non-Bahraini: 0				
Exon 13	c.1733_1734delTA [p.Leu578ArgfsX10]	1	0 (0%)	Bahraini: 1	1 (0.9%)	1 (1.1%)	-	1
				Non-Bahraini: 0				
Exon 20	c.3196C>T [R1066C; p.Arg1066Cys]	1	0 (0%)	Bahraini: 1	1 (0.9%)	1 (1.1%)	-	1
				Non-Bahraini: 0				
Intron 17	c.2909-15T>G [3041-15 T>G]	1	0 (0%)	Bahraini: 0	1 (0.9%)	1 (1.1%)	-	1
				Non-Bahraini: 1 Jordanian				
Exon 13	c.1736A>G [D579G; p.Asp579Gly]	1	0 (0%)	Bahraini: 0	1 (0.9%)	1 (1.1%)	-	1
				Non-Bahraini: 1 Jordanian				
Total		71	36 (50.7%)	71	112	92	31	30

^**a**^pwCF with compound heterozygous mutations are counted twice

^**b**^Number of consanguineous (from 1st cousin marriages) pwCF carrying the *CFTR* allele /number of total pwCF with the allele

^**c**^Frequency of *CFTR* alleles is calculated by dividing the total number of alleles for each mutation by the total number of CF alleles in the pwCF population (112)

^**d**^The number of *CFTR* alleles excludes repeats of the same mutation within CF siblings. The frequency is calculated by dividing the resultant number of *CFTR* alleles for each mutation by the total number of alleles after exclusion (92)

^e^The homozygous and compound heterozygote *CFTR* allele count was made after the exclusion of allele repeats within siblings

In the case of multiple CF siblings in a studied family with identical genotypes (i.e. overall 18 siblings from 8 families; with 7 families having two, whilst the remaining family had 4 siblings with CF), variants present in homozygosity or heterozygosity in *trans,* we considered only one of the affected children for an aggregate overview of their CF population frequencies. Similarly, in these families, the consanguinity rate was calculated by considering only one affected child. Hence, the resulting overall percentage of mutations in the Bahraini population (and its subpopulations) is extrapolated as the number of given *CFTR* variants divided by the total of CF alleles of the entire cohort under examination. The percentage of CF alleles in the examined families is calculated as the number of families with the CF-causing variants divided by the total number of CF alleles in the entire CF population. For the *CFTR* variant nomenclature, we used the recommendation of the locus-specific CFTR1 consortium database and those of the Human Genome Variation Society (den Dunnen et al. 2016). However, in the Discussion section, we resorted to the still more widely utilised ‘legacy nomenclature’ (in brackets). Variant pathogenicity was assessed according to the ‘Clinical and Functional Translation of CFTR’ database (CFTR2; www.cftr2.org) and CFTR France (https://cftr.iurc.montp.inserm.fr/cftr) and where applicable we utilised the VarSome portal (https://varsome.com/). Finally, we also checked the ClinVar database (https://www.ncbi.nlm.nih.gov/clinvar/) for variants not reported to the CFTR1 database.

In the remaining 35 incompletely genotyped pwCF, dry blood spot samples were drawn (i.e. ‘Guthrie cards’) and sent for genotyping at the Motol University Hospital (Prague; Czechia), based on unrestricted academic collaboration. From the methodological point of view, first, the 50 most common *CFTR* mutations in European-derived populations using the Elucigene^Tm^ CF-EU2 assay (Elucigene Diagnostics, United Kingdom), followed by massively parallel sequencing of the entire *CFTR* coding region, adjacent splice site junctions, selected CNVs and several introns using a locus-specific library preparation assay (CFTR NGS assay^Tm^; Devyser, Sweden; www.devyser.com) on the MiSeq System^Tm^ next-generation sequencing platform (Illumina, USA; www.Illumina.com) were examined. Bioinformatic analysis was carried out using the comprehensive SOPHiA DDM Platform for Hereditary Disorders^Tm^ (www.sophiagenetics.com/technology/sophia-ddm-for-genomics), where applicable positive cases were confirmed by targeted Sanger DNA sequencing on ABI 3130xl DNA Analyser^Tm^ (ThermoFisher, USA; www.thermofisher.com; protocol available upon request). In addition, multiplex ligation-dependent probe amplification (MLPA) analysis of intra-*CFTR* rearrangements and CNVs was performed by the SALSA MLPA P091 CFTR Assay^Tm^ followed by an analysis of raw data on the proprietary software Coffalyser.Net^Tm^ (MRC-Holland, The Netherlands; www.MRCholland.com) to complement analysis carried out within the CFTR NGS assay^Tm^ which was validated primarily on European-derived populations.

Ethical approval for this study was obtained from the SMC Ethical Committee and the Secondary Health Care Research Committee representing the Ministry of Health of Bahrain. Informed consent was taken from all pwCF or their fiduciaries. To increase confidentiality, pseudonymisation was used for their identification within commercial and academic laboratories. Statistical analysis of descriptive clinical and laboratory data was carried out using the MS Excel statistical functionality.

## Results

### Incidence of the disease

The estimated mean incidence of CF within our fully genotyped cohort of pwCF with the classical form of CF within the two-decade-long study period is (56/27) 2.07 live births/year, i.e. approximately 1 per 9,880 live births.

### Demographic data

The retrospective analysis identified a total of 56 pwCF with the classical form of the disease from all over Bahrain, whereby 42 pwCf are of Bahraini origin. Fourtenn are of foreign origin, comprising 5 cases of Syrian, 3 of Jordanian, 3 of Pakistani and 1 of each Palestinian, Indian and Yemeni. A total of 27 pwCF originated from 10 large CF families where we documented consanguinity, comprising 7 Bahraini families, and one each from a Syrian, a Pakistani and a Jordanian family. The remaining 29 pwCF are independent cases with a negative family history of the disease, comprising 24 Bahraini and 1 of each Syrian, Palestinian, Indian, Yemini and Jordanian origin.

The overall consanguinity rate in the Bahraini cohort is 42.9% (15/35 – counting only pwCF from families with multiple siblings as specified above), all due to 1st cousin marriages. Interestingly, although not statistically significant due to small numbers, the rate of 1st cousin marriages is markedly higher in the CF population of foreign origin (72.7%–8/11; Table 1).

Overall, the ratio of male to female pwCF is 32:24 (i.e. ratio of 1.3:1.0). The median age at diagnosis is 6 months (range 1–108 months). pwCF of Bahraini origin is generally older (mean age at follow-up is 12.9 ± 6.9 years) and 8 pwCF from the total cohort were adults over 18 years of age (17.0%). In the overall study cohort, 47 (83.9%) survived and 9 (16.1%) died, with an average age of death 12.9 ± 4.3 years.

Another specific aspect of the Bahraini CF population is the presence of comorbidity with the classical form of sickle cell disease (MIM: 603903; ORPHA:232; SCD) in 2/56 (3.6%) of all pwCF, with the two cases being consanguineous and of Bahraini origin (Table 1).

### Clinical and laboratory features

Faltering growth represents the most common initial diagnostic symptom observed in 29/48 (60.4%) of all pwCF (hardcopy archive data were not available for 8 older cases), most of them falling under the 5th percentile in terms of their weight as compared with their age and sex-matched controls as per current reference growth charts Centers for Disease Control and Prevention 2017). The mean sweat chloride concentration is 110.6 mM (range 73–135 mM) for the entire cohort using sweat conductivity measurements (ELITechGroup; USA - https://www.elitechgroup.com/).

The rates of pancreatic insufficiency (generally over 96.4%; 54/56) and chronic *P. aeruginosa* bronchial colonisation (over 87.8%; in a total of 43/49 pwCF where sputum culture and/or deep tracheal aspirations could be performed) are particularly high in all pwCF. Additionally, three colonised pwCF suffered from methicillin-resistant *S. aureus*, one of whom died. Similarly, the entire CF population is undernourished in 41/48 (85.4%) of all cases as expressed by their body mass index values (Table 1). Lastly, 5/56 (8.9%) of all pwCF have CF-related diabetes mellitus (CFRD) which is associated with higher mortality (3/5 cases as of the publication submission; detailed data for aforementioned clinical/laboratory parameters are available upon request).

### Sequencing of the entire *CFTR* coding region

A total of 21 already genotyped pwCF had both CF-causing mutations identified in *trans* (Table 2), whilst 35 cases remained with one of both variants unidentified and were genotyped in this study as outlined in the Patients and Methods section. The analysis of 56 pwCF revealed 18 different *CFTR* mutations, of which 15 have not been detected before in Bahrain. The rate of homozygosity (62/92 = 67.4%), i.e. according to the applied methodology 56 × 2–20 alleles is present in multiple siblings (Table 2). The most common mutations in the overall CF population of Bahrain are c.1911delG [2043delG], c.2988+1G>A [3120+1G>A], c.2989-1G>A [3121-1G>A], c.3909C>G [N1303K], and c.1521_1523delCTT [p.Phe508del; F508del] in order of decreasing frequency, i.e. excluding multiple affected siblings from a given family. In the group of Bahraini origin, 2043delG, 3120+1G >A and 3121-1G>A equally make up the most common mutations, each accounting for 15 alleles after the exclusion of siblings, followed by N1303K.

The group of non-Bahraini origin has 2043delG as the most common mutation, which is concentrated in all pwCF of Syrian origin within our cohort, whilst *CFTR* mutations of Indian, Jordanian, Pakistani, Palestinian and Yemeni origin are listed in Table 2. Two *CFTR* alleles, which were not reported in the CFTR1 database but are reported in the ClinVar database were found for the first time in Bahrain—c.743+2T>C (Accession number: VCV000645792.1) in homozygosity and the c.1733_1734delTA (VCV000623296.2) [p.Leu578ArgfsX10] / 3120+1G>A in compound heterozygosity. In both instances, their linkage phase is based on family segregation studies.

Finally, in cases that did not comply with consensus diagnostic criteria and are thus considered separately from Tables 2 and 3, variant c.173A>G (p.Asp58Gly) [D58G] in homozygosity, including a variant c.389T>C (p.Leu130Pro)[L130P]/wt were detected (ref. Discussion section). Variant pathogenicity using VarSome and in the CFTR France database, both indicate that the D58G variant is likely pathogenic (PP3, PM1, PM2 and variant of unknown significance (VUS4), respectively), whilst in the CFTR1 database, it was previously reported in Tunisia in an individual suffering from the congenital bilateral agenesis of vas deferens (CBAVD; MIM: 277,180). The L130P variant is a VUS according to VarSome and is absent in the CFTR2 and CFTR France databases. For illustration, the allelic composition of detected compound heterozygote genotypes is presented in Supplementary Table 3.

**Table 3:** Allelic composition of all compound heterozygote pwCF within the Bahraini cohort (Supplementary table)

Allele 1	Allele 2	Number of pwCF with this allele composition
c.1911delG [2043delG;Gln637fsX662]	c.1647 T>G [S549R(T>G); p.Ser549Arg]	1
c.1911delG [2043delG;Gln637fsX662]	c.3196C>T [R1066C; p.Arg1066Cys]	1
c.1911delG [2043delG;Gln637fsX662]	c.1418delG [1548delG; p.Gly473GlufsTer54]	1
c.1911delG [2043delG;Gln637fsX662]	c.2989-1G>A [3121-1G>A; IVS18-1G>A]	1
c.1911delG [2043delG;Gln637fsX662]	c.2988+1G>A [3120+1G>A; IVS18+1G>A]	3
c.2988+1G>A [3120+1G>A; IVS18+1G>A]	c.416A>T [H139L; p.His139Leu]	1
c.2988+1G>A [3120+1G>A; IVS18+1G>A]	c.1733_1734delTA [p.Leu578ArgfsX10]	1
c.2988+1G>A [3120+1G>A; IVS18+1G>A]	c.1397C>G [S466X (TAG); p.Ser466X]	1
c.2988+1G>A [3120+1G>A; IVS18+1G>A]	c.2989-1G>A [3121-1G>A; IVS18-1G>A]	1
c.2989-1G>A [3121-1G >A; IVS18-1G>A]	c.1397C>G [S466X (TAG); p.Ser466X]	1
c.1521_1523delCTT [p.Phe508del]	c.1647T>G [S549R(T>G); p.Ser549Arg]	1
c.223C>T [R75X; p.Arg75Ter]	c.274-1G>A [406-1G>A]	1
c.2909-15T>G [3041-15 T>G]	c.1736A>G [D579G; p.Asp579Gly]	1

## Discussion

This study presents an overview of the *CFTR* mutation distribution in a representative cohort of 56 Bahraini-resident pwCF, including multiple siblings in individual families (i.e. proportionally representing the entire resident and non-resident population) and originating from all regions of the country. Furthermore, this study outlines the basic demographics and clinical characteristics of this CF cohort, and to date is one of the most comprehensive reports on a Middle Eastern CF population together with a recently published Saudi review (Banjar et al. 2020). We also carried out incidence estimates accounting for the three-decade-long study perspective and provided a basis for the introduction of variant-specific therapies, such as CFTRm. Our study could serve as a basis for other analyses of this kind in non-European populations with a higher degree of consanguinity. In this respect, population characteristics of the cohort under study are relevant since particularly in non-European populations, associated with generally lower prevalence of the p.Phe508del major European-derived mutation that is the primary driver of historic heterozygote advantage. The higher degree of consanguinity which increases recessive disorders such as CF and the higher granularity in terms of pwCF ethnicity are also relevant as we have documented for the reported cases of Iraqi, Kurdish or Syrian origin.

### Prevalence and incidence of the disease

Compared to the previously recorded incidence of 1 in 5800 live births (Al-Mahroos 1998), the incidence observed in this study (1 per 9880 live births) is lower than that of other Middle Eastern reports (Banjar and Angyalosi 2015). However, it should be noted that our value is based on the study’s cohort only, which includes pwCF who comply with consensus diagnostic criteria for the classical form of CF and who underwent complete analysis of the entire *CFTR* coding region. This higher “stringency” of case ascertainment could explain why our study demonstrated one of the lowest rates of CF incidence in the Middle East. Although our incidence estimate could be biased by consanguinity, it needs to be noted that all Bahraini residents get full access to CF care from the national healthcare system. Thus, we do not expect a large amount of underdiagnosed pwCF and the two-decade-long study period evens out eventual year-to-year fluctuations of CF incidence. Nevertheless, we would like to emphasise that this estimate should still be carefully considered since it could be biassed due to the relatively small number of cases.

### Demographic data

A previous research paper from 2002 studying CF in Bahrain recorded the overall consanguinity within their cohort as being 77% (Eskandarani 2002). Intriguingly, in this study, we observed a marked decrease in the consanguinity rate/ 1st cousin marriages with the total being approximately 50% within our cohort. Herewith, pwCF of Bahraini origin currently have a consanguinity rate of 42.9% and those of non-Bahraini origin at 72.7%. We presume that the observed decrease in consanguinity rate is related to our larger cohort group, improved public health awareness leading to a decreased rate of 1st cousin marriages, together with our increased efficiency at detecting pwCF even amongst families not known for having the disease. In addition, the previous report very likely suffered from an ascertainment bias where a subset of the general population with higher consanguinity rates was more likely detected by the healthcare system by being affected with more clinically severe recessive disorders, such as CF and/or SCD. Nonetheless, although the prevalence of SCD is 1% in the general Bahraini population (Al Arrayed and Haites 1995), we found it to be 3.6% in this study.

This implies that consanguinity, albeit decreased, still plays a role in CF prevalence. pwCF of non-Bahraini–Syrian origin showed higher rates of consanguinity within our cohort (80%) (Othman & Saadat 2009), compared to the 1st cousin marriage rate of the general population in Syria (28.7%). The same phenomenon is seen within all other pwCF of non-Bahraini origin within our cohort (Afzal et al. 1994; Jurdi and Saxena 2003; Bittles 2002; Sirdah 2014; Islam et al. 2018). The consanguinity rate is significant in both Bahrain and Saudi Arabia pwCF. However, Saudi Arabia’s consanguinity rate in this cohort is higher (85%) than that of Bahrain’s CF cohort (50.0%) (Banjar et al. 2020).

For comparisons of basic demographic data in this study, we used a large retrospective study carried out in Saudi Arabia which analysed data between January 1, 1998, and December 31, 2017, as well as the European Cystic Fibrosis Society Patient Registry 2020 Annual Data Report (ECFSPR). In this respect, Bahrain’s median age of diagnosis (6 months) is significantly earlier than in Saudi Arabia (10.2 months). However, the median age of diagnosis in the ECFSPR database (3.6 months) is earlier than in both Bahrain and Saudi Arabia. pwCF recorded in the ECFSPR were also more likely to be ≥ 18 years old (53.1%) compared to our cohort (17.0%), and ECFSPR cases had a significantly higher mean age at death (Bahrain: 12.9 years, ECFSPR: 32.0 years). However, it should be noted that the mean age at death within our cohort is shifted due to a single outlier who passed away at the age of 4 years. When that case is excluded, the mean age at death is 16.1 years (Banjar et al. 2020; European Cystic Fibrosis Society 2020). Previous reports from Bahrain described the mean age of pwCF at follow-up as ranging from 4 months to 14 years (Eskandarani 2002), whereas the current range is 1.7–28.7 years, which shows improved care and is reflected by an increasing proportion of adults (Table 1). There wasn´t any significant difference between males and females in our cohort in terms of their survival (data available upon request).

### Clinical and laboratory features

In terms of clinical presentation, both Bahrain (96.4%) and Saudi Arabia (85%) cases have significantly high rates of pancreatic insufficiency, but lower rates of CFRD (Bahrain: 8.9%, Saudi Arabia: 12%) compared to ECFSPR (26.9%). The difference in terms of CFRD prevalence could reflect that our pwCF are generally younger. Bahrain’s pwCF are more likely to have *P. aeruginosa* respiratory colonisation (87.8%) compared to the Saudi cohort (approx. 60%) and ECFSPR cases (11.7%). pwCF living with lung transplants were more common amongst ECFSPR patients (5.7%) than our cases (1.8%) (Banjar et al. 2020; European Cystic Fibrosis Society Patient Registry 2020).

Another specific aspect of the Bahraini population is the presence of comorbidity with SCD in 3.6% of all pwCF (Table 1). These cases primarily come from consanguineous families; hence, these have an increased probability of recessive disorders in a single affected individual. The higher prevalence of SCD in the Bahraini population is a considerable factor as well (see above). Neither the ECFSPR nor the most recent Saudi study reported SCD and such observations remain rare worldwide (Banjar 2003; Banjar et al. 2020; European Cystic Fibrosis Society 2020). The first SCD case is 10 years old with a body mass index (BMI) of 12.6 kg/m2, pancreatic insufficiency, severe bronchiectasis and chronic *P. aeruginosa* colonisation. The second case is 11 years old with a BMI of 14.8 kg/m2, pancreatic insufficiency and severe lung disease with chronic *P. aeruginosa* colonisation, indicating a more severe course of CF in both instances.

### Sequencing of the entire *CFTR* coding region and adjacent splice sites

Bahrain’s geographic location has led to substantial historic population migrations from the regions surrounding the Arabian Gulf, including Africa (Al-Snan, 2020). In addition, the generally high rate of consanguinity (see above) leads to a higher frequency of *CFTR* mutations observed in homozygosity (67.4%). There are similarities with Saudi Arabia with regard to the types of mutations observed (see further) with most of them being in a homozygous constitution. However, we observed a higher rate of compound heterozygosity (32.6%) compared to Saudi Arabia (7.4%) (Banjar et al. 2020).

Comprehensive analysis of the entire *CFTR* coding region by next-generation sequencing, including adjacent splice sites and eight selected intronic regions (as per manufacturer´s specification; Devyser.com) in a representative cohort of resident pwCF in Bahrain revealed 18 different *CFTR* mutations. The five most common alleles in the overall cohort are 2043delG, 3120+1G>A, 3121-1G>A, N1303K and p.PheF508del in decreasing order of their frequency. The ranking is slightly different in pwCF of Bahraini origin, where the most common mutations are equally shared between 2043delG, 3120+1G>A and 3121-1G>A (all being present at 13 alleles each), with N1303K being the second most common. Cases of non-Bahraini origin have 2043delG as the most common allele, with all pwCF carrying this allele being of Syrian origin.

The most common mutation in Bahrain is 2043delG accounting for 22.8% of CF alleles (after exclusion of siblings). This mutation has been previously documented in Bahrain at a high frequency of 30.8%, which given the small size of the study population could reflect an ascertainment bias (Eskandarani 2002). This mutation is being increasingly documented in other countries from the Arabian Gulf region, such as documented e.g. in Saudi Arabia (5.2%), Iran (12.5%) and Lebanon (2.5%) (Banjar et al. 2020; Dooki et al. 2015; Farra et al. 2010). It is notable that all pwCF of Syrian origin from settlements along the Euphrates River likely originate from a single large family and therefore only have this mutation (Table 2).

The second most common mutation is 3120+1G>A accounting for 16.3% of all CF alleles. This mutation is also referred to as the ‘African’ CF mutation and has been reported on 9–14% of African American CF chromosomes (Macek et al. 1997; Padoa et al. 1999). Kambouris et al. found that screening for five *CFTR* mutations, one of which is this allele could achieve a 60% detection rate in Arab CF populations (Kambouris et al. 2000). This mutation was previously reported in both Bahrain (3.8% of all alleles, but at a small and not representative cohort likely underestimating its overall frequency), Saudi Arabia (11% in its Eastern provinces) and Oman (8.7%) (Eskandarani 2002; Banjar et al. 2020; Al-Kindy et al. 2014). El-Harith et al. documented that this mutation in homozygosity is associated with early disease presentation, faltering growth, severe CF lung disease, *P. aeruginosa* lung colonisation and pancreatic insufficiency (el-Harith et al. 1997). Accordingly, our pwCF who mostly have this allele in compound heterozygosity, presented with faltering growth, are pancreatic insufficient and colonised with *P. aeruginosa*. The compound heterozygous pwCF with a novel allele in trans- c.1733_1734delTA [p.Leu578ArgfsX10] also has the classical form of CF.

The third most common mutation 3121–1G>A (13.4%) has not been reported in Bahraini CF pwCF before, however, was reported in southern Iraqi CF families of declared Jewish origin. The authors noted its carrier frequency of 1 in 68.5 in the Jewish population of Basra and added it to the screening protocol in ‘Oriental Jews’ (Reish et al. 2009). Similarly, another study found this allele in Jewish pwCF from Kurdistan (Quint et al. 2005). This mutation was found also in non-Jewish pwCF, as it has also been reported in two Iranian cases from the Khuzestan province in Southwestern Iran (Alibakhshi et al. 2008). Hence, there is likely a “mutation hot-spot” in the western regions surrounding the Arabian Gulf and from there on it is found in the respective diaspora living in Western Europe and North America.

The fourth most common mutation is N1303K, accounting for 13.0% of CF alleles. It occurs in one large family of Bahraini origin as well as in a case of Palestinian origin. Previous reports in Bahrain indicated its frequency at 7.7% (Eskandarani 2002), whilst in Saudi Arabia, its frequency is between approximately 2–3% (Banjar et al. 2020; el-Harith et al. 1997). This mutation is considered as being an ‘Eastern Mediterranean’ mutation due to its highest worldwide frequency in Lebanon (27%), which might explain its presence in a case of Palestinian origin within our cohort (Farra et al. 2010; Farhat et al. 2015).

The fifth most common CF-causing mutation in Bahrain is p.Phe508del, which is the most common CF mutation in the European-derived populations, with a frequency of approximately 70% amongst American, Canadian and Northern European Caucasians (Morral et al. 1994). However, the mutation only has an allele frequency of 7.6% from all CF alleles in Bahrain. Ranking as the fifth most common mutation in this study, the mutation appeared in 6 Bahraini pwCF, 4 of whom came from the same family, as well as 1 pwCF of Pakistani origin. This mutation is more common in Iran, e.g. 18.1% in Tehran and 17.5% in its Northern parts (Alibakhshi et al. 2008; Oskooei et al. 2013). Accordingly, this mutation was previously found in the Bahraini population of Persian origin and is the third most common in Saudi Arabia (11.4%) (Eskandarani 2002; Banjar et al. 2020).

The c.743+2T>C (described as likely pathogenic in the ClinVar database) was found for the first time in two Bahraini siblings who are homozygous for this allele since their parents are related. They are 6 (with sweat chloride concentrations of 85 mM) and 13 years (94 mM) old. Interestingly, they happen to be the only pancreatic-sufficient cases in our cohort and one of the few pwCF without chronic *P. aeruginosa* colonisation. Both alleles, c.743+2T>C and c.1733_1734delTA (p.Leu578fs; originally detected in Omani CF patient), were reported by us to the CFTR1 database.

### CFTR-related disorders and unclear diagnoses

The D58G was found in homozygosity in an 8-year-old male (from a 2nd cousin family) and only one of his repeated sweat chloride concentrations was in the borderline range of 40 mM. He was identified as a neonate via raised immunoreactive trypsinogen (IRT) levels detected in a private setting through an ad hoc neonatal screening procedure. He was indicated to SMC for genetic testing, whereby we found this mutation. This pwCF does not have symptoms of classical CF and is being longitudinally monitored at SMC. The D58G allele was previously identified in a male of Tunisian origin, who is residing in France and was incidentally diagnosed with CBAVD. The Tunisian case bears the D58G in compound heterozygosity with the N1303K mutation (Claustres et al. 2000). Due to the young age of our case, we so far have not had a chance to investigate the possibility of him having CBAVD. Finally, for the c.389T>C [L130P], there is no further clinical/laboratory except for reported low birth weight and bowel obstruction since they abruptly died in the early neonatal period. Thus, we could not complete *CFTR* genotyping due to the lack of available biospecimen.

### CFTR modulator therapy

In terms of variant-specific therapy currently, five pwCF already receive CFTRm, four having p.Phe508del in homozygosity, including one compound heterozygote for the S549R(T>G) mutation. Apart from standard symptomatic therapy, one successful bilateral lung transplantation was carried out in a 3121–1G>A homozygote in July 2018.

In conclusion, our data provide a strong basis for the improvement of *CFTR* genetic diagnostics in classical forms of CF by achieving an almost 100% population-based mutation detection rate, the eventual introduction of CF neonatal screening in the country using e.g. an IRT-DNA strategy and provides a basis for the introduction of other forms of targeted therapies beyond CFTRm in indicated cases.

## Data Availability

All unpublished data are available upon request.

## Abbreviations

BMI: Body Mass Index
CBAVD: Congenital Bilateral Absence of the Vas Deferens
CF: Cystic Fibrosis
CFRD: CF-related diabetes mellitus
CFTR: Cystic Fibrosis Transmembrane Conductance Regulator
CFTRm: CFTR modulator therapy
ECFSPR: European Cystic Fibrosis Society Patient Registry
IRT: Immunoreactive Trypsinogen
*P. aeruginosa*: *Pseudomonas aeruginosa*
pwCF: People with CF
*S. aureus*: *Staphylococcus aureus*
SCD: Sickle Cell Disease
SMC: Salmaniya Medical Complex
VUS: Variant of unknown significance
